# Prospective randomised unblinded comparison of sputum viscosity for three methods of saline nebulisation in mechanically ventilated patients: A pilot study protocol

**DOI:** 10.1371/journal.pone.0290033

**Published:** 2023-08-17

**Authors:** Andrew Arnott, Robert Hart, Scott McQueen, Malcolm Watson, Malcolm Sim

**Affiliations:** Critical Care Department, Queen Elizabeth University Hospital, Glasgow, United Kingdom; CHU Nantes, FRANCE

## Abstract

**Introduction:**

Heat and moisture exchanger (HME) filters are commonly used as passive circuit humidifiers during mechanical ventilation, however, are only ~80% efficient. As a result, patients that undergo mechanical ventilation in critical care with HME filter circuits will be exposed to partial airway humidification. This is associated with detrimental effects including increased secretion load which has been shown to be an independent predictor of failed extubation. Nebulised normal saline is commonly utilised to supplement circuit humidification in ventilated patients with high secretion loads, although there are no randomised control trials evaluating its use. Novel vibrating mesh nebulisers generate a fine aerosol resulting in deeper lung penetration, potentially offering a more effective means of nebulisation in comparison to jet nebulisers. The primary aim of this study is to compare the viscosity of respiratory secretions after treatment with nebulised normal saline administered via vibrating mesh nebuliser or jet nebuliser.

**Methods and analysis:**

This randomised controlled trial is enrolling 60 mechanically ventilated adult critical care patients breathing on HME filter circuits with high secretion loads. Recruited patients will be randomised to receive nebulised saline via 3 modalities: 1) Continuous vibrating mesh nebuliser; 2) Intermittent vibrating mesh nebuliser or 3) Intermittent jet nebuliser. Over the 72-hr study period, the patients’ sputum viscosity (measured using a validated qualitative sputum assessment tool) and physiological parameters will be recorded by an unblinded assessor. A median reduction in secretion viscosity of ≥0.5 on the qualitative sputum assessment score will be deemed as a clinically significant improvement between treatment groups at analysis.

**Discussion:**

At the conclusion of this trial, we will provisionally determine if nebulised normal saline administered via vibrating mesh nebulisation is superior to traditional jet nebulisation in terms of reduced respiratory secretion viscosity in intubated patients. Results from this pilot study will provide information to power a definitive clinical study.

**Trial registration:**

ClinicalTrails.Gov Registry (NCT05635903).

## Introduction

Critically unwell mechanically ventilated patients have decreased ability to effectively clear their own secretions. This is due to multiple factors such as impaired cough, low conscious level and respiratory muscle weakness. Patients with intercurrent pulmonary infection and aspiration are also likely to have an increased overall secretion load, compounding the problem of poor clearance [1]. In critical illness, a high secretion load is a major risk factor for tracheal extubation failure and the need for reintubation [2]. Failed extubation has also been shown to be an independent predictor of poor outcome regardless of underlying disease severity [3], and is associated with prolonged mechanical ventilation and significantly increased total health-care cost [4]. As a result, effective secretion management is key to facilitating early extubation, reducing critical care stay and improving outcomes.

During respiration the upper airways heat and humidify inhaled gases to 37°C with a relative humidity of 100% (absolute humidity of 44mg/L H_2_O) [5]. The extent of airway water losses during this process is determined by the temperature and humidity of the inhaled gas, and reduces as temperature and humidity increase (S1 Table). In temperate climates (25°C, 50% relative humidity, absolute humidity 11.5mg/L) ~246ml/day of water is lost through normal respiration (assuming a tidal volume of 500ml and a respiratory rate of 14). Under the same conditions, inhalation of dehumidified gas (e.g. relative humidity 10%, absolute humidity 2.3mg/L) increases airway water losses by almost 30% (~315ml/day). During mechanical ventilation (via endotracheal tube (ETT) or tracheostomy) the upper airway is bypassed and the body’s ability to heat and humidify inhaled gases is impaired. Dehumidified gas has numerous detrimental effects on the airways distal to the bronchioles and the function of the mucociliary elevator, including increased mucus viscosity, decreased ciliary function and tracheal inflammation [6]. The adverse effects of dry gas inhalation have also been shown to increase the risk of post-operative respiratory complications in surgical patients [7], highlighting the importance of complete inspired gas humidification during prolonged ventilation in critical care.

Heat and moisture exchanger (HME) filters are a passive humidification method commonly utilised during mechanical ventilation. Placed between the patient and the Y-connector, HME filters cause exhaled water vapours to condense on and heat a specially designed membrane [8]. HME filters function by evaporating the retained water and heating the gases during inspiration and returning them to the patient’s airways [5]. HME filters are a cheap and effective way of maintaining circuit humidity and have the additional benefit of acting as an antimicrobial filter. Despite this, even modern HMEs are only ~80% efficient at returning exhaled vapours to the circuit [9, 10]. They can also become clogged in patients with high secretion loads leading to increased airflow resistance and difficulties ventilating [11]. Sole reliance on HME filters to maintain circuit humidity may expose patients to the detrimental effects of inadequate airway humidification and mucociliary dysfunction. As a result, airway humidification is often augmented with nebulised saline.

Nebulisers are active humidification devices that can be used intermittently or continuously to increase the baseline circuit humidity established by HME filters. All nebulisers work by vaporising a body of liquid into a fine aerosolised mist that can be delivered deep into the lungs [12]. In critical care nebulisers are commonly used to nebulise normal saline (typically 0.9% normal saline) for ventilated patients with troublesome secretion loads. The clinical benefit of nebulised normal saline is hypothesised to be reduced secretion viscosity as a result of complete humification of gases, facilitating clearance during tracheal suctioning. Several studies in non-critically ill patients with obstructive lung diseases have demonstrated nebulised normal saline as an effective sputum inducer [13–15], although there are no randomised control trials (RCTs) evaluating the use of normal saline in ventilated patients.

Although there is no robust data to confirm the safety of nebulised normal saline, the use of nebulised therapies in critical care is widespread and well-tolerated. Ehrmann et al. [16] conducted an international multi-centre prospective observational study in 2808 critical care patients finding that <1% experienced side-effects effects as a results of nebulisation. The majority of these side-effects were mild (i.e. tachycardia, hypertension), with only 3 reports of bronchospasm all associated with nebulised colistin. Lyu et al. [17] observed similar results in a multi-centre prospective observational study in 1006 Chinese critical care patients, with mild side effects reported in 8% of patients receiving nebulised therapies and no serious adverse events. Very serious adverse events (i.e. cardiac arrest, pneumothorax) reported in other case studies are rare and all associated with blocked expiratory filters [18–20]. Other technical factors must also be taken into consideration for the safe delivery of nebulisers in invasively ventilated patients. Circuit disconnection for nebuliser attachment can resulting in lung de-recruitment, and jet nebulisers reliant on external flow can alter inspired oxygen concentrations and increase airways pressures in ventilated circuits [21, 22]. As a result, vigilance is required during nebuliser delivery as ventilator settings may need to be adjusted to ensure safe delivery.

Modern vibrating mesh nebulisers (VMN) generate a precisely sized slow velocity aerosol that reduces circuit condensation and increases pulmonary delivery [21, 23, 24]. They aerosolise solutions via a rapidly vibrating piezo element [25] and their clinical superiority over conventional jet nebulisers (JN) has been demonstrated in multiple studies [26–29]. VMN also have multiple practical advantages over JN and ultrasonic nebulisers, such as faster treatment times, lower residual volumes and in-line usage and are widely regarded as the future of nebulisation [21, 24]. VMN may offer a more effective route for the administration of nebulised normal saline in critical care secretion management compared to traditional JN.

### Study rationale

Despite the widespread use of nebulised normal saline for secretion management in critical care during mechanical ventilation, there are no RCTs evaluating its use. Furthermore, there are no RCTs comparing the effectiveness of normal saline delivery via VMN or JN for mechanically ventilated patients with high secretion loads. A greater understanding of the effects of nebuliser device choice (i.e. VMN or JN) and dosing regimen on secretion viscosity are required to improve critical care secretion management in HME filter circuits.

### Aims

The primary aim of this study is to compare the effect of normal saline nebulisation via VMN and JN on secretion viscosity in ventilated critical care patients breathing on HME filter circuits.

Secondary aims will include analysis of:

- Total secretion volume
- Respiratory physiology (i.e. work of breathing, airway resistance)
- Frequency of any additional nebulised therapies administered over study period (normal saline or other nebulised therapeutics)
- Qualitative bedside nurse feedback questionnaire (e.g. ease of sampling)
- Frequency of required HME filter changes
- Frequency of adverse events

We hypothesise that nebulised normal saline will: 1) reduce secretion viscosity and improve respiratory physiology in ventilated critical care patients on HME filter circuits due to improved circuit humidification; and 2) that VMN will be superior to JN in-terms of reducing secretion viscosity due to increased pulmonary drug delivery.

## Materials and methods

### Study design

Prospective unblinded single-centre randomised control trial

### Participating centres

Queen Elizabeth University Hospital, Glasgow, United Kingdom

### Participant selection

#### Inclusion criteria

- Patients aged 18–80 years at time of recruitment
- Ventilated via an ETT or tracheostomy with an HME filter in the circuit
- Secretion load defined as patient requiring suctioning at least 2 times in the 6 hours prior to recruitment
- Sputum viscosity with grades 1 to 3 pourability defined by the Qualitative Sputum Assessment Tool (QSAT)
- No nebulised saline administration in the 6 hours prior to recruitment
- Patient likely to remain ventilated (via an ETT or tracheostomy) for at least 3 days in the opinion of the treating clinician

#### Exclusion criteria

- Pregnancy
- Pulmonary embolus
- Heart Failure (New York Heart Association Functional Classification Grade III/IV)
- Clinical evidence of frank pulmonary oedema
- Cardiovascular instability (systolic blood pressure ≤75 or heart rate ≥140)
- COVID-19 infection
- Chronic disease affecting mucociliary clearance (i.e. cystic fibrosis)

### Sample size rationale

A total of 60 patients will be recruited to the study (20 in each of the 3 treatment groups):

1. Continuous VMN: continuous nebulisation of 0.9% normal saline using the Aerogen Solo Nebuliser (50mls/24hrs via a syringe feed set).
2. Intermittent VMN: intermittent nebulisation of 0.9% normal saline using the Aerogen Solo Nebuliser (5mls 6 hourly).
3. Intermittent JN: intermittent nebulisation of 0.9% normal saline using the Intersurgical Cirrus 2 self-sealing Jet Nebuliser (5mls 6 hourly)

### Primary endpoint

Viscosity of respiratory secretions as assessed by the QSAT pourability score which has been previously validated in the literature [30–32].

### Secondary endpoints

- Volume of secretions
- Work of breathing
- Airway resistance
- Frequency of any additional nebulised therapies administered over study period (normal saline or other nebulised therapeutics)
- Ease of sampling in opinion of bedside nurse (0–10 analogue scale)
- Frequency of required HME filter changes
- Length of time on ventilator
- Length of stay in critical care
- Critical care mortality
- Frequency of adverse events

### Study procedures

#### Consent

**Fig 1:**
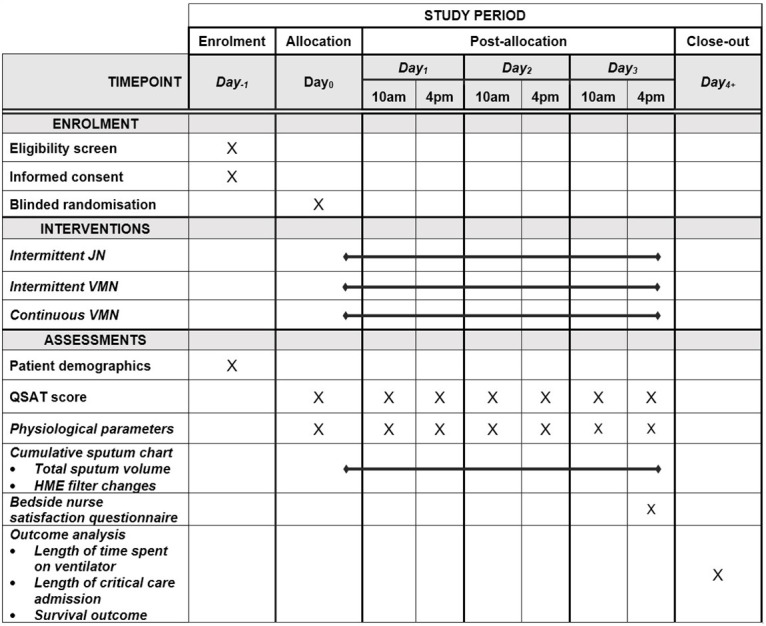
SPIRIT schedule showing study procedure.

The SPIRIT schedule showing study procedure is illustrated in Fig 1. Patients eligible for recruitment will be identified and approached by a member of the critical care research team. Eligibility will be assessed against the inclusion and exclusion criteria by a doctor with an up-to-date Good Clinical Practice certificate who is on the study delegation log. Informed written consent will be obtained for all patients recruited to this study. As eligible patients will lack capacity due to sedation and mechanical ventilation, consent will be sought from a legal representative/nearest relative [in accordance with the ‘Adults with Incapacity (Scotland) Act 2000’]. The legal representative will be provided with a written information sheet and a member of the research team will answer any questions prior to consent being taken. The legal representative may withdraw the patient at any stage. Retrospective consent with be sought for patients recruited to the trial who later regain capacity. If the patient never regains capacity or dies, then they will remain in the study and their data will be included in the final analysis. In instances where no legal representative/nearest relative is identified patients will not be enrolled.

#### QSAT analysis and data collection

**Fig 2:**
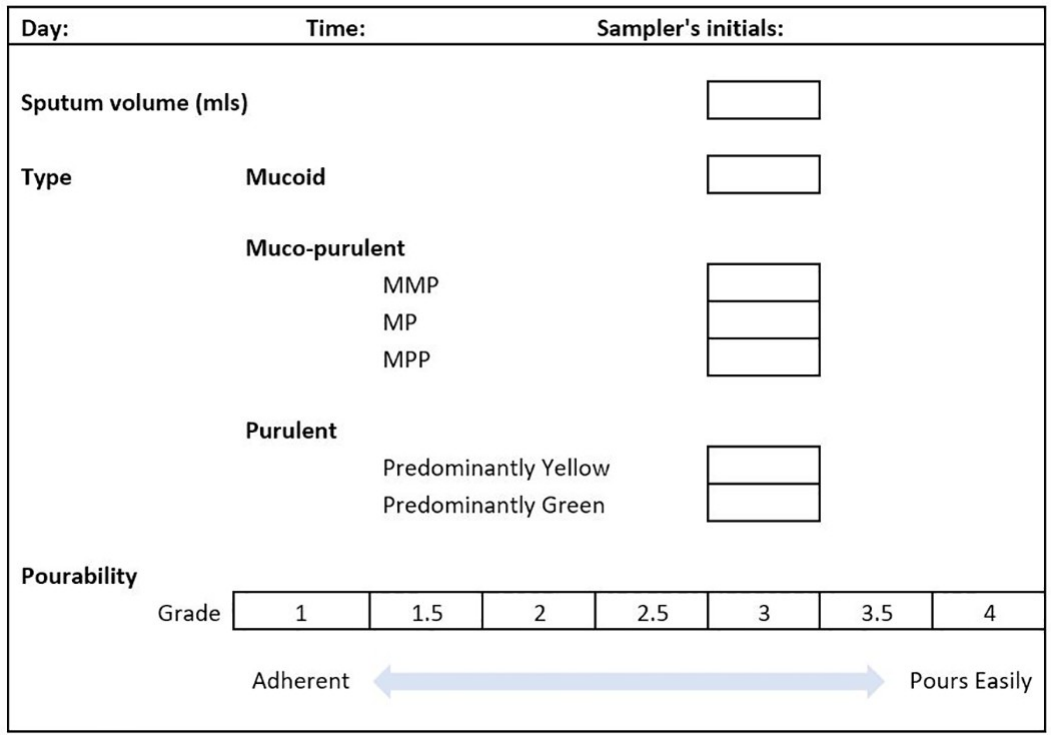
Qualitative Sputum Assessment Tool (QSAT) used for sputum analysis.

When a patient fulfils the entry criteria and is consented to participate, they will be blindly randomised (via shuffled opaque sealed envelopes corresponding to study number) by a member of research team to one of the 3 treatments: (1) Continuous VMN (continuous nebulisation of 0.9% normal saline using the Aerogen Solo Nebuliser. Placed between the ETT and HME filter. 50mls/24h via a syringe feed set); (2) Intermittent VMN (intermittent nebulisation of 0.9% normal saline using the Aerogen Solo Nebuliser. Placed between the ETT and HME filter. 5mls, 6 hourly); or (3) Intermittent JN (intermittent nebulisation of 0.9% normal saline using the Intersurgical Cirrus 2 self-sealing Jet Nebuliser. Placed between the ETT and HME, driven by 6L/min walled oxygen. 5mls, 6 hourly). A cumulative sputum volume chart will be given to the patient’s bedside nurse to record cumulative sputum volumes over the 72hr duration of the study. This will allow the recording of secretions on routine suction in addition to any HME filter changes. The patient’s QSAT score (Fig 2) and physiological parameters will be recorded on day 0 (time of recruitment prior to nebulise therapy commencing) and at 10:00 and 16:00 on day 1, 2 and 3 of the study. To ensure continuity, QSAT analysis will always be carried out by an unblinded member of the delegation log (critical care specialist physiotherapists and physicians) who have been trained in sputum analysis. Following completion of the trial, nebulisation may be continued/discontinued as determined by the clinical team. The bedside nurse will also be asked to fill in a nurse satisfaction questionnaire regarding the nebuliser device used. If at any point, there is clinical concern from the treating clinician the trial will be stopped.

### Clinical data

Relevant patient data will be extracted from clinical notes by the local research team using standardised data collection forms. Completed forms will be transcribed to a password protected electronic spreadsheet kept on a secure server. Paper forms will then be stored securely in the site file within a locked office at the participating site. All data will be anonymised at site and a unique study number will be allocated to each patient. The following data will be collected: age, sex, respiratory and cardiovascular parameters, length of time spent on ventilator, length of critical care stay and survival outcome.

### Planned analysis

This is a prospective single-centre randomised control study and a total of 20 patients in each treatment group is adequate to allow determination of the primary and secondary endpoints [33]. This will provide information to power a definitive clinical study. As the QSAT score ranges from 1 to 4 in increments of 0.5, the distribution of values of change ranges from -3 to +3 in 0.5 increments. Therefore, the QSAT score can be treated as a continuous measurement. A median reduction in sputum viscosity of ≥0.5 on the QSAT pourability scale will be determined as a clinical improvement between groups. With 20 subjects we estimate an 80% power to detect a difference between any 2 groups of 0.503 per standard deviation (effect size = 0.503) for the change in QSAT score. This assumes a level of significance (p-value) of 0.0167 taking into account the use of 3 comparisons:

1. Intermittent JN vs. Continuous VMN
2. Intermittent JN vs. Intermittent VMN
3. Continuous VMN vs. Intermittent VMN

[The statistical analysis and powering of this study were based on the advice of Dr Michele Robertson, Senior Statistician at The Robertson Centre for Biostatistics (University of Glasgow)]

### Patient confidentiality

Patient confidentiality will be maintained throughout the study as per the Data Protection Act (2018). Recruited patients will be assigned a unique study number. All data collection sheets, study reports and communications regarding the study will identify patients by study number and initials only.

### Adverse event reporting

Any adverse event or adverse device event will be recorded on a case report form and reported to the patients’ assigned consultant and the principal investigator (PI). All serious adverse events will be similarly recorded and immediately flagged to the patients’ assigned consultant, the PI, the research and development office and the assigned ethics committee for further investigation. Adverse event analysis with take place as part of this study’s secondary outcome analysis.

### Protocol deviation reporting

A variation of the approved protocol will be considered a protocol deviation. Any protocol deviations will be recorded on case report forms and reported to the sponsor.

### Ethics approval

This study was approved by the NHS Greater Glasgow & Clyde Research & Development Ethics Committee (reference 19/SS/0116) and will be carried out in accordance with the World Medical Associated Declaration of Helsinki (1964) and its revisions (Tokyo (1975), Venice (1983), Hong Kong (1989), South Africa (1996) and Edinburgh (2000)).

### Dissemination

Authorisation for all publications will be sought from the study PI and will be reviewed by the sponsor prior to publication. The results of completed analysis will be published in a peer-reviewed scientific journal and at national/international conferences.

### Protocol number

This protocol represents the updated protocol version 6 (dated 19^th^ November 2019) for the study (S1 File). Any future protocol amendments will be re-discussed with the study sponsor, ethics committee and updated on the ClinicalTrials.Gov registry prior to implementation.

### Patient and public involvement

Patients/the public were not involved in any aspect of study design, recruitment or future dissemination of results.

### Trial status

The trial is currently open for recruitment.

## Discussion

Effective secretion management in mechanically ventilated critical care patients is essential to improve likelihood of successful extubation and outcome. Nebulised saline is commonly utilised to help with secretion management in HME circuits but no RCTs have been conducted to examine its use. As a result, the optimum delivery device and dosing regimen for normal saline administration to reduce secretion viscosity remains undetermined. This pilot study aims to establish an understanding of the changes in sputum viscosity after continuous and intermittent normal saline nebulisation using VMN and JN. This is with the goal of powering a definitive study to improve secretion management in mechanically ventilated critical care patients breathing on HME filters.

### Study limitations

1. A blinded study was infeasible as all members of the critical care research team are involved in the clinical care of enrolled patients out-with their research role. Patients randomised to each arm are also identifiable to researchers collecting sputum samples as the Aerogen Solo Nebuliser and Intersurgical Cirrus 2 self-sealing Jet Nebuliser T-piece remain in the circuit throughout the study period.
2. COVID-19 patients were excluded from this study because the initial protocol was designed at the start of the pandemic when there were safety concerns regarding fugitive emissions and aerosol generation during nebulisation.
3. Studies have shown that VMN have reduced residual medication volumes in comparison to jet nebulisers [21, 34, 35]. As 5ml 0.9% normal saline was used in all treatment arms, patients randomised to VMN with potentially receive more normal saline throughout the study period. This will be taken into account during analysis of the pilot data.

## Supporting information

S1 Table Estimated airway water losses (ml/day) during normal respiration at different temperatures and relative humidities. Assumes a tidal volume of 500ml, a respiratory rate of 14 and a 25% return of water vapour transferred to inspired air during exhalation as described in the literature [36]. Values for absolute humidity obtained from the Transport Informations Service [37].
(PDF)

S1 File Study protocol (version 6). (PDF)

S2 File SPIRIT checklist. (PDF)

## Abbreviations

HME: Heat and moisture exchanger
JN: Jet nebuliser
PI: Principal investigator
QSAT: Qualitative sputum assessment tool
RCT: Randomised control trial
VMN: Vibrating mesh nebuliser
