# Supplementary material for: Prospective randomised unblinded comparison of sputum viscosity for three methods of saline nebulisation in mechanically ventilated patients: A pilot study protocol
Source: PLoS One. 2023 Aug 17;18(8):e0290033. doi: 10.1371/journal.pone.0290033 (PMC10434882; doi:10.1371/journal.pone.0290033)
Supplement: S1 Table — Assumes a tidal volume of 500ml, a respiratory rate of 14 and a 25% return of water vapour transferred to inspired air during exhalation as described in the literature [36]. Values for absolute humidity obtained from the Transport Informations Service [37]. (PDF) [file pone.0290033.s001.pdf]

S1 Table. Estimated airway water losses (ml/day) during normal respiration at different temperatures and relative humidities.

		Relative humidity of inspired air									
		10%	20%	30%	40%	50%	60%	70%	80%	90%	100%
Temperature	**35°C**	302	273	243	213	183	153	123	93	64	33
	**30°C**	310	287	264	241	218	195	172	149	126	103
	**25°C**	315	298	280	263	246	228	211	194	176	159
	**20°C**	320	306	293	280	267	254	241	228	215	202

Assumes a tidal volume of 500ml, a respiratory rate of 14 and a 25% return of water vapour transferred to inspired air during exhalation as described in the literature.[36] Values for absolute humidity obtained from the Transport Informations Service.[37]
