# Supplementary material for: Prospective randomised unblinded comparison of sputum viscosity for three methods of saline nebulisation in mechanically ventilated patients: A pilot study protocol
Source: PLoS One. 2023 Aug 17;18(8):e0290033. doi: 10.1371/journal.pone.0290033 (PMC10434882; doi:10.1371/journal.pone.0290033)
Supplement: S1 File — (PDF) [file pone.0290033.s002.pdf]

Study Title:

Can continuous or intermittent normal saline nebulisation via a vibrating mesh nebuliser or intermittent normal saline via a standard jet nebuliser improve the lung physiology and secretion viscosity in mechanically ventilated patients with a Heat Moisture Exchange (HME)?

**Protocol Version:**	**6**
**Date:**	**19/11/19**
**REC Reference Number:**	**19/SS/0116**
**IRAS Number:**	**271083**
**Clinical trial.gov Number:**	**NCT05635903**
**Sponsor's Protocol Number:**	**GN18RM440**
**Sponsor:**	**NHS Greater Glasgow & Clyde**
**Funder:**	**Aerogen Ltd,<br>Galway Business Park,<br>Dangan, Galway**

Amendment number	Date	Protocol version

This study will be performed according to the Research Governance Framework for Health and Community Care (Second edition, 2006) and WORLD MEDICAL ASSOCIATION DECLARATION OF HELSINKI Ethical Principles for Medical Research Involving Human Subjects 1964 (as amended).

CONTACTS

Chief Investigator

Dr. Malcolm Sim
Consultant in Anaesthesia and Intensive Care Medicine
Queen Elizabeth University Hospital
1345 Govan Road
Glasgow
G51 4TF
Tel: 0141 452 3033
Fax: N/A
E-mail: malcolm.sim@ggc.scot.nhs.uk

Co-investigator

Dr. Malcolm Watson
Consultant in Anaesthesia
Queen Elizabeth University Hospital
1345 Govan Road
Glasgow
G51 4TF

Tel: 0141 452 3034
Fax: N/A
E-mail: malcolm.watson@ggc.scot.nhs.uk

Project Manager/Study Co-ordinator

Sophie Kennedy-Hay
Senior Research Nurse
Glasgow Clinical Research Facility
5th Floor, Institute of Neurological Sciences
Queen Elizabeth University Hospital
1345 Govan Road
Glasgow G51 4TF
01412327600

Tel: 0141 232 7600
Fax: N/A
E-mail: Sophie.KennedyHay@ggc.scot.nhs.uk

Study Statistician

Mrs. Michele Robertson
R1122F Level 11
Robertson Centre
Boyd Orr Building
Glasgow
G12 8QQ

Tel: 0141 330 3163
Fax: N/A
E-mail: Michele.Robertson@glasgow.ac.uk

Sponsor – NHS Greater Glasgow and Clyde health board

Sponsor's representative
Dr Maureen Travers
Research Co-ordinator
R&D Management Office,
Clinical Research & Development
Dykebar hospital
Grahamston Road,
Paisley
PA2 7DE

E-mail: Maureen.Travers@ggc.scot.nhs.uk
Telephone: 0141 314 4012

Funding Body

Aerogen Ltd.
Galway Business Park
Dangan
Galway
Ireland
H91 HE94

PROTOCOL APPROVAL

Can continuous or intermittent normal saline nebulisation via a vibrating mesh nebuliser or intermittent normal saline via a standard jet nebuliser improve the lung physiology and secretion viscosity in mechanically ventilated patients with a Heat Moisture Exchange (HME) filter in the circuit?

Chief Investigator **Dr. Malcolm Sim**
Consultant in Anaesthesia and Intensive Care Medicine
Critical Care
Queen Elizabeth University Hospital
1345 Govan Road
Glasgow
G51 4TF

Signature:

Date: <Date>

Sponsor's representative **Dr Maureen Travers**
Research Co-ordinator
NHS Greater Glasgow & Clyde
Research and Development Management Office
Dykebar hospital
Grahamston Road,
Paisley
PA2 7DE

Signature:

Date: <Date>

TABLE OF CONTENTS

CONTACTS......2
TABLE OF CONTENTS......5
GLOSSARY OF TERMS......7
ABBREVIATIONS......7
STUDY SYNOPSIS......8
STUDY FLOW CHART......10
SCHEDULE OF ASSESSMENTS......12
1. INTRODUCTION 13
1.1 Background 13
1.2 Rationale...... 13
1.3 Study hypothesis 13
2. STUDY OBJECTIVES 14
3. STUDY DESIGN 14
3.1 Study Population 14
3.3 Exclusion criteria...... 15
3.4 Identification of participants and consent 15
3.5 Withdrawal of subjects...... 16
4. TRIAL PROCEDURES...... 17
4.1 Study schedule – Please see study flow chart 17
4.2 Study Outcome Measures...... 17
4.2.1 Primary Outcome Measure 17
4.2.2 Secondary Outcome Measure 17
4.3 Laboratory Tests...... 17
5. ASSESSMENT OF SAFETY...... 18
6. PHARMACOVIGILANCE...... 19
6.1 Definitions of adverse events...... 19
6.2 Serious Adverse Event (SAE) 19
6.3 Serious Adverse Device Events......19
6.4 Reporting and documentation of complications, and adverse device effects......20
6.5 Reporting of serious adverse events to the Sponsor......20
6.6 Reporting of related and unexpected events to the REC......21
7. STATISTICS AND DATA ANALYSIS 21
7.1 Statistical analysis plan 21
7.2 Primary efficacy analysis 21
7.3 Secondary efficacy analysis 21
7. 4 Safety analysis 21
7.5 Software for statistical analysis...... 20
7. 6 Sample size...... 22
7.7 Management and delivery 22
8. STUDY CLOSURE / DEFINITION OF END OF TRIAL 23
9. DATA HANDLING 23
9.1 Randomisation **...... 23**
9.2 Case Report Forms / Electronic Data Record...... 23
9.3 Record Retention...... 23
10. TRIAL MANAGEMENT...... 24
10.1 Routine management of trial: Trial Management Group 24
11. STUDY MONITORING/AUDITING...... 25
12. PROTOCOL AMENDMENTS...... 26
13. ETHICAL CONSIDERATIONS...... 27
13.1 Ethical conduct of the study 27

14. INSURANCE AND INDEMNITY 28
15. FUNDING 28
16. ANNUAL REPORTS 28
17. DISSEMINATION OF FINDINGS 28
18. REFERENCES 28

GLOSSARY OF TERMS

ABBREVIATIONS

AE	*Adverse event*
REC	*Research Ethics Committee*
SAE	*Serious Adverse Event*

STUDY SYNOPSIS

Title of Study:	Can continuous or intermittent normal saline nebulisation via a vibrating mesh nebuliser or intermittent normal saline via a standard jet nebuliser improve the lung physiology and secretion viscosity in mechanically ventilated patients with a Heat Moisture Exchange (HME) filter in the circuit?
Study Centre:	Queen Elizabeth University Hospital
Duration of Study:	12 months
Primary Objective:	Comparison of the viscosity of respiratory sections produced using a vibrating mesh nebuliser or a traditional jet nebuliser.
Secondary Objectives:	• Volume of secretions<br>• Work of breathing<br>• Airway resistance<br>• Number of additional nebulised doses of saline or other drugs administered during the study period<br>• Nurse opinion of ease of sampling<br>• Frequency of HME filter change
Primary Endpoint:	Viscosity of respiratory sections (assessed by the Qualitative Sputum Assessment Tool).
Rationale:	Nebulisation improves secretion management and respiratory physiology by reducing viscosity of respiratory secretions.
Methodology:	Prospective randomised pilot study.
Sample Size:	60 patients
Screening:	Patients ventilated via an endotracheal tube or tracheostomy in the Intensive Care Units of the Queen Elizabeth University Hospital and identified at the daily meeting between physiotherapists and nursing staff as having a secretion load.
Registration/Randomisation:	Computer generated randomisation
Main Inclusion Criteria:	• Patient aged 18-80 years at time of recruitment to study<br>• Ventilated via an endotracheal tube or tracheostomy with an HME filter in the circuit<br>• Secretion load defined as patient requiring suctioning of secretions at least 2 times in the 6 hours prior to recruitment.

	• Sputum viscosity grade 1 to 3 pourability in the Qualitative Sputum Assessment tool. (See below)<br>• Not yet received saline nebulisation in the previous 6 hours prior to recruitment<br>• Likely to be ventilated via an endotracheal tube or tracheostomy for at least 3 days in the opinion of the treating clinician
Main Exclusion Criteria:	• Pregnancy<br>• Pulmonary embolus<br>• Heart Failure (NYHA Grade III/IV)<br>• Clinical evidence of frank pulmonary oedema<br>• Cardiovascular instability (systolic BP ≤75 or heart rate ≥140)
Duration of Treatment:	Up to 72 hours
Product, Dose, Modes of Administration:	Continuous nebulisation of 0.9% normal saline using the Aerogen Solo Nebuliser (50mls/24h via a syringe feed set) OR<br><br>Intermittent nebulisation of 0.9% normal saline using the Aerogen Solo Nebuliser (5mls, 6 hourly) OR<br><br>Intermittent standard nebulisation using the Intersurgical Cirrus 2 self sealing Jet Nebuliser (5 mls, 6 hourly)
Statistical Analysis:	Comparison of the difference in the viscosity of respiratory sections compared using Kruskall Wallis test with **SPSS**, **release 19.0** for Windows; **SPSS**, Chicago, IL

STUDY FLOW CHART

Patient screened in the Intensive Care Unit of the Queen Elizabeth University Hospital

Inclusion Criteria:

Patient aged 18-80 years at time of recruitment to study
Ventilated via an endotracheal tube or tracheostomy with an HME filter in the circuit
Secretion load defined as patient requiring suctioning to remove respiratory secretions at least 2 times in the 6 hours prior to recruitment
Sputum viscosity with grades 1 to 3 pourability in the Qualitative Sputum Assessment tool
Not yet received saline nebulisation in the 6 hours prior to recruitment
Likely to be ventilated via an endotracheal tube or tracheostomy for at least 3 days in the opinion of the treating clinician

Exclusion Criteria:
Pregnancy
Pulmonary embolus
Heart Failure (NYHA Grade III/IV)
Clinical evidence of frank pulmonary oedema

Initial sample of sputum obtained and Qualitative Sputum Assessment undertaken as follows:

**Volume (mls)**			
**Type**	M, Mucoid (no evidence of pus)		
	P, Purulent (uniformly purulent) (Colour also recorded)	Predominantly yellow	
		Predominantly green	
	MP, Mucopurulent (mixed) (Divided into 3 grades depending whether pus or mucus is predominant)	MMP	
		MP	
		MPP	
**Pourability**	Grade 1 - adheres closely to the container		
	Grade ½(1.5)		
	Grade 2 - moves slowly with gravity		
	Grade 2/3(2.5)		
	Grade 3 – slides in one mass		
	Grade ¾(3.5)		
	Grade 4 – pours easily but may have viscid particles		

Randomisation

Continuous nebulisation of 0.9% saline via the Aerogen Solo (50mls/24h via a syringe feed set) for up to 3 days

(20 patients)

Intermittent nebulisation of 0.9% saline via the Aerogen Solo (5mls 6 hourly) for up to 3 days

(20 patients)

Intermittent standard nebulisation of 0.9% saline using the Intersurgical Cirrus 2 self-sealing Jet Nebuliser (5mls 6 hourly) for up to 3 days

(20 patients)

Patients will be suctioned a minimum of every 4 hours and the volume of sputum recorded

Patients can be suctioned in between times as required if required clinically (and the volume of sputum recorded)

The Quantitative Sputum Assessment will be undertaken by a dedicated group of physiotherapists (to ensure consistent analysis). This will occur once in the morning (as close to 10am as possible) and once in the afternoon (as close to 4pm as possible), ideally at least 1 hour after the end of a nebulisation in the intermittent groups.

Secondary endpoints recorded including:
Respiratory physiological parameters will be recorded.
Number of additional nebulised doses of saline or other drugs administered during the study period, ease of sampling, frequency of HME filter change.
Length of stay in ICU, Total duration of ventilation and ICU mortality

SAFETY POINTS

If at any point there is clinical concern the treating clinician can stop the study at their discretion.

In the Intermittent Groups the patient can receive more frequent nebulisation if required clinically.

Additional drugs can be nebulised at any point and in any group in addition to 0.9% Saline at any point if there is clinical concern.

SCHEDULE OF ASSESSMENTS

Study Procedure	Visit 1	Visit2	Visit3	Visit 4	Visit 5	Visit 6	Visit 7
Obtain Informed Consent from patient or relative incapacity form	√						
Review Inclusion/Exclusion Criteria	√						
Recruit patient if appropriate and undertake study over 3days	√						
Day 1- monitor secretions, undertake Qualitative Sputum Analysis and respiratory physiology		√	√				
Day 2 - monitor secretions, undertake Qualitative Sputum Analysis and respiratory physiology				√	√		
Day 3 - monitor secretions, undertake Qualitative Sputum Analysis and respiratory physiology						√	√

1. INTRODUCTION

1.1 Background

Critically unwell patients in Intensive Care have a decreased ability to effectively clear secretions. This is often multifactorial and can include respiratory muscle weakness, impaired cough and low conscious level. In addition the amount of secretions can be higher than normal due to intercurrent infection or aspiration.[1] High secretion load is a major risk factor in the failure of tracheal extubation failure and requirement for reintubation.[2] Extubation failure is a predictor of poor outcome independent of the severity of the underlying illness.[3] Nebulisation of isotonic saline can be employed to manage secretions by reducing the secretion viscosity and facilitating clearance of respiratory sections during tracheal suction.

We are testing the hypothesis that regular (or continuous) small doses of nebulised normal saline will benefit lung physiology in mechanically ventilated patients (with a heat and moisture exchanger filter in the circuit). This practice of additional nebulisation is commonplace in ICUs when secretions management is problematic.

If totally dry gases were inspired and fully saturated gases exhaled the total water loss from ventilation at rest would be about 300 ml/day in the average adult. Bypassing the nose with an endotracheal tube and not humidifying gases causes maximal losses. This is mitigated by using a heat and moisture exchange filter (HME) in the circuit. The standard HME is 80 % efficient then the water loss from the airway will be around 60ml/day. Patients with underlying lung issues or difficult to manage secretions may benefit from optimised airway hydration and improved lung physiology.

Standard jet nebulisers have been the mainstay of respiratory section management therapy for in critical care since the early 1990's. A more recent development has been the vibrating mesh nebuliser. There is evidence of improved humidification and reduced water particle size and theoretically better transfer to the distal airways.

1.2 Rationale

The vibrating mesh nebuliser (Aerogen technology) may be superior to standard nebuliser technology.

1.3 Study hypothesis

Improved secretion management with reduced tenacity of respiratory sections and potentially improved lung physiology secondary to improved humidification or reduced size of nebulised particles?

2. STUDY OBJECTIVES

Primary Endpoint

- Pourability of respiratory secretions (As assessed by the Qualitative Sputum Assessment Tool)

  (The QSA score will assess quantity, quality/stickiness/density and colour/appearance of secretions and is described and validated in the literature[3,4])

Secondary endpoints

- Volume of secretions (increased or decreased may be beneficial)
- Work of breathing
- Airway resistance
- Number of additional nebulised doses of saline or other drugs administered during the study period
- Ease of sampling, in opinion of treating nurse
- Frequency of requiring changing the HME filter
- Length of time on ventilator
- Length of stay in ICU
- ICU Mortality

3. STUDY DESIGN

3.1 Study Population

A total of 60 patients will be recruited to the study. Each patient will be randomised to receive:

Continuous nebulisation of 0.9% normal saline using the Aerogen Solo Nebuliser (50mls/24h via a syringe feed set) OR

Intermittent nebulisation of 0.9% normal saline using the Aerogen Solo Nebuliser (5mls, 6 hourly) OR

Intermittent standard nebulisation of 0.9% normal saline using the Intersurgical Cirrus 2 self-sealing Jet Nebuliser (5 mls, 6 hourly)

3.2 Inclusion criteria

- Patient aged 18-80 years at time of recruitment to study
- Ventilated via an endotracheal tube or tracheostomy with an HME filter in the circuit

- Secretion load defined as patient requiring suctioning at least 2 times in the 6 hours prior to recruitment
- Sputum viscosity with grades 1 to 3 pourability in the Qualitative Sputum Assessment tool
- Not yet received saline nebulisation in the 6 hours prior to recruitment
- Likely to be ventilated via an endotracheal tube or tracheostomy for at least 3 days in the opinion of the treating clinician

3.3 Exclusion criteria

- Pregnancy
- Pulmonary embolus
- Heart Failure (NYHA Grade III/IV)
- Clinical evidence of frank pulmonary oedema
- Cardiovascular instability (systolic BP ≤75 or heart rate ≥140)

3.4 Identification of participants and consent

Patients with acute respiratory failure will be screened in the Intensive Care Units of the Queen Elizabeth university hospital.

i) Patient with capacity:

- The patient will be provided with a written ‘Patient Information Sheet’.
- A member of the research team with relevant GCP training will provide verbal information and answer any questions.
- If the patient chooses to be enrolled in the study, they will sign a ‘Patient Consent Form’.
- The patient may withdraw consent at any stage (as explicitly stated in the ‘Patient Information Sheet’).

ii) Patient without capacity, when a legal representative is immediately available:

- When a patient does not have capacity the research team will attempt to identify a legal representative [in accordance with the ‘Adults with Incapacity (Scotland) Act 2000’ (Scotland)]
- The legal representative will be provided with a written ‘Legal Representative Information Sheet’.
- A member of the research team with relevant GCP training will provide verbal information and answer any questions.
- The legal representative will be asked to use their knowledge of the patient’s beliefs to advise the research team as to whether they feel the patient would choose to enrol in the study.
- When the legal representative feels the patient would have chosen to enrol in the study, they will be asked to sign a consent form on behalf of the incapacitated adult.

- The legal representative may withdraw the patient at any stage (as explicitly stated in the 'Legal Representative Information Sheet').
- In the event that the patient regains capacity, the research team will speak to the patient at the earliest opportunity and ask the patient to provide retrospective consent; if the patient chooses to continue to be part of the study they will sign a 'Consent Form to continue'; if the patient gives consent to continue, they are free to withdraw their consent at any time; if the patient does not wish to consent, they will be withdrawn from the study.
- In the event that the patient never regains capacity or dies then he/she will remain in the study and their data will be included in the final analysis.

iii) Patient without capacity, when a Legal Representative is not immediately available, and no appropriate person is identified:

- When a patient does not have capacity and the research team are unable to identify or contact an appropriate legal representative, (Welfare Attorney, Welfare Guardian or Nearest Relative) the patient should not be enrolled into the study.

3.5 Withdrawal of subjects

Patients will be withdrawn if they cannot tolerate nebulisation via either method or if there is concern by the treating clinician at any point during the study.

4 Trial procedures

4.1 Study schedule – Please see study flow chart

Visit 1: Screening

- Assess if patient meets the entry criteria
- Give verbal and written information either to patient if they have capacity or legal representative.

Visit 2: Randomisation and undertaking of study

Patient randomised to Continuous nebulisation of 0.9% normal saline using the Aerogen Solo Nebuliser (50mls) OR Intermittent nebulisation of 0.9% normal saline using the Aerogen Solo Nebuliser (5mls, 6 hourly) OR Intermittent standard nebulisation of 0.9% normal saline using the Intersurgical Cirrus 2 self-sealing Jet Nebuliser (5 mls, 6 hourly)

4.2 Study Outcome Measures

4.2.1 Primary Outcome Measure

- Median change of all viscosity (pourability) of respiratory secretions as assessed by a dedicated team or physiotherapists using the Qualitative Sputum Assessment Tool Scores taken at 10:00 and 16:00 while the patient is recruited to the study

4.2.2 Secondary Outcome Measure

- Volume of secretions (increased or decreased may be beneficial)
- Work of breathing
- Airway resistance
- Number of additional nebulised doses of saline or other drugs administered during the study period
- Ease of sampling, in opinion of treating nurse
- Frequency of requiring changing the HME filter
- Length of time on ventilator
- Length of stay in ICU
- ICU Mortality

4.3 Laboratory Tests

The patient will have no additional blood tests as part of the study.

5. ASSESSMENT OF SAFETY

Throughout the duration of the study period the recruited patients will be closely monitored by a Intensive Care Nurses and critical care physicians. They will have continuous monitoring of their oxygen saturations (SpO_2), heart rate, with regular monitoring of blood pressure. As part of standard care patients will have regular monitoring of their ventilator parameters including the tidal volume being delivered and airway pressures.

The treating clinician can stop the nebulisation if at any point they have clinical concern and institute any rescue therapy deemed necessary.

6. PHARMACOVIGILANCE

6.1 Definitions of adverse events

Adverse Event (AE)

AEs can be defined as any untoward medical occurrence in a patient or other clinical investigation participant taking part in a trial of a medical device, which does not necessarily have to have a causal relationship with the device under investigation.

An AE can therefore be any unfavourable and unintended sign (including an abnormal laboratory finding), symptom or disease temporally associated with the use of the device, whether or not considered related to the device.

Adverse events that are study related and not complications of nebulisation are hard to identify. Anticipated adverse events/complications that can occur with any nebulisation are bronchospasm, accumulation of fluid in the circuit, feeling of chest tightness.

These will all be documented on AE report forms and reported to the Chief Investigator.

6.2 Serious Adverse Event (SAE)

SAE is an adverse event that

- Led to death
- Led to serious deterioration in the health of the subject that:
  - Resulted in a life-threatening illness or injury
  - Resulted in a permanent impairment of a body structure or a body function
  - Required in-patient hospitalisation or prolongation of existing hospitalisation
  - Resulted in medical or surgical intervention to prevent permanent impairment to a body structure or a body function

SAEs are not expected due to the trial, but the investigators are aware that SAEs may occur from nebulisation.

6.3 Serious Adverse Device Events (SADE)

A serious adverse device effect (SADE) is any untoward medical occurrence seen in a patient that can be attributed wholly or partly to the device which resulted in any characteristics of a serious adverse event.
SADE is also any event that may have led to these consequences if suitable action had not been taken or intervention had not been made or if circumstances had been less opportune. All cases judged by either the reporting medically qualified professional or the sponsor.

6.4 Reporting and documentation of complications, and adverse device effects.

Complications and Adverse Events will be documented on Adverse Event Report forms for the duration of the investigation. Furthermore, the outcome of such complications will be documented and any changes in outcome updated during the study. Each complication and adverse event must be assessed for seriousness, causality and expectedness against the expected complications resulting from both the surgical procedure and the use of the medical device(s). All complications will be reported to the Chief Investigator.

6.5 Reporting of serious adverse events to the Sponsor.

The following events are considered reportable to the sponsor.

- Any Serious Adverse Event that is considered related to the surgical procedure, trial specific procedures, or the medical device(s) that is considered unexpected by the Chief Investigator or their delegate.
- Any device related serious adverse event that led or may have led to one of the following outcomes:
    - The death of a patient
    - A serious deterioration in the health of a patient.

  A serious deterioration in health may include (non-exhaustive):

  a) Life threatening illness

  b) Permanent impairment of a body function or permanent damage to a body structure

  c) A condition necessitating medical/surgical intervention to prevent a) or b)

  d) Foetal distress or death, or any congenital anomalies or birth defects.

All SAEs meeting the above criteria must be reported to the Pharmacovigilance Office immediately (within 24 hours) using the SAE form for a CE Marked Medical Device. The SAE form should be completed and signed by appropriately delegated staff. The form should be faxed or e-mailed to the PV Office (pharmacovig@glasgowctu.org) and a copy placed in the Study Site File. If necessary, a verbal report can be given by contacting the PV Office on 0141 330 4744. This must be followed up as soon as possible with a signed written (or electronic) report.

If all of the required information is not available at the time of initial reporting, the CI (or designee) must ensure that any missing information is forwarded to the PV Office as soon as this becomes available. The report should indicate that this information is follow-up information for a previously reported event.

6.6 Reporting of related and unexpected events to the REC

The Sponsor will report all RUSAEs to the ethics committee within 15 days of the PV office becoming aware of the event, via the 'report of serious adverse event form' for non-CTIMPs published on the Health Research Authority web site. http://www.hra.nhs.uk/documents/2015/02/safety-report-form-non-ctimp.docx. The form should be completed in typescript and signed by the Chief Investigator.

7. STATISTICS AND DATA ANALYSIS

7.1 Statistical analysis plan

A total of 60 patients will allow us to determine the distribution of the primary and secondary outcome measures[6] which will provide information to power a further clinical study. Since this is a pilot study, we are not powered to formally compare differences between the treatment groups.

7.2 Primary efficacy analysis

A median reduction in the sputum viscosity by 0.5 on a pourability scale for respiratory sections between the three humidification methods tested.

7.3 Secondary efficacy analysis

- Volume of secretions (increased or decreased may be beneficial)
- Work of breathing
- Airway resistance
- Number of additional nebulised doses of saline or other drugs administered during the study period
- Ease of sampling, in opinion of treating nurse
- Frequency of requiring changing the HME filter
- Length of time on ventilator
- Length of stay in ICU
- ICU Mortality

7. 4 Safety analysis

The safety data (adverse events) – both numbers of subjects and events – will be summarised by randomised group and overall using descriptive statistics. No formal statistical tests comparing the randomised groups will be pre-specified. Adverse event will be reported to GGC and the local ethic committee annually and required by Scottish Research Governance Standards.

7.5 Software for statistical analysis

The statistical software to be used will be SPSS, release 19.0 for Windows; SPSS, Chicago, IL

7. 6 Sample size

This is a pilot feasibility study and a total of 20 patients in each group is adequate to provide information to power a definitive study[6]. The chosen samples sizes can be further justified as follows:

We will determine if the primary end point (viscosity even you are looking at the change in the score from baseline to final measurement, assuming the score is 1 to 4 in 0.5 increments at each time point, the distribution in values of change could range from -3 to +3 in 0.5 increments. This can therefore be treated as a continuous measurement. With 20 subjects we estimate an 80% power to detect a difference between any 2 groups of 0.503 per standard deviation (effect size=0.503) for the change in score. This assumes a significance level (p value) of 0.0167 to take into account that we would use 3 comparisons:

1. (Intermittent standard nebulisation vs. Continuous Aerogen nebulisation,
2. Intermittent standard nebulisation vs. Intermittent Aerogen nebulisation,
3. Continuous Aerogen nebulisation vs. Intermittent Aerogen nebulisation)

The statistically analysis and powering of this study were based on advice for Dr Michele Robertson senior statistician the Robertson Centre, 24th July 2019.

7.7 Management and delivery

The Robertson Centre for Biostatistics, part of the Glasgow Clinical Trials Unit, a fully registered UK CRN Clinical Trials Unit, will analyse the trial data. All statistical analyses will be conducted according to a pre-specified Statistical Analysis Plan and advice from the Robertson Centre, Glasgow University.

8.0 STUDY CLOSURE / DEFINITION OF END OF TRIAL

The study will end when the Chief Investigator and Sponsor agree that one or more of the following situations applies:

i. The planned sample size has been achieved;
ii. There is insufficient funding to support further recruitment, and no reasonable prospect of additional support being obtained;
iii. New information makes it inappropriate to continue to randomise patients to one or other arm of the trial;
iv. Recruitment is so poor that completion of the trial cannot reasonably be anticipated.

9. Data Handling

9.1 Randomisation

Randomisation to the 3 treatment groups will be by means of a web-based randomisation system.

9.2 Case Report Forms / Electronic Data Record

A written case report form (CRF) will be used to collect study data. The CRF has been developed by the chief investigator, with only authorised site-specific personnel able to make entries or amendments to their patients' data. It will be the investigator's responsibility to ensure completion and to review and approve all data captured in the CRF.

9.3 Record Retention

The chief investigator will keep records, including the identity of all participating subjects, all original signed informed consent forms, serious adverse event forms, source documents for a minimum of 1 year after the last patient is recruited to the study. The anonymised study data will be retained for a total of 5 years.

10.0 TRIAL MANAGEMENT

10.1 Routine management of trial: Trial Management Group

The trial will be coordinated from The Queen Elizabeth university hospital by Dr Malcolm Sim and Dr Malcolm Watson. The role of the group will be to monitor all aspects of the conduct and progress of the trial, ensure that the protocol is adhered to and take appropriate action to safeguard participants and the quality of the trial itself.

11. STUDY MONITORING/AUDITING

This study will be risk assessed by NHS Greater Glasgow and Clyde Health Board Research and Development department and audit of this study will be determined by the results of the risk assessment.

12. PROTOCOL AMENDMENTS

Any change in the study protocol will require an amendment. Any proposed protocol amendments will be initiated by the CI following discussion with the sponsor and any required amendment forms will be submitted to the ethics committee and sponsor. The CI will liaise with study sponsor to determine whether an amendment is non-substantial or substantial. All amended versions of the protocol will be signed by the CI and Sponsor representative. A favourable opinion/approval must be sought from the original reviewing REC and Research and Development (R&D) office(s) before the amended protocol can be implemented.

13. ETHICAL CONSIDERATIONS

13.1 Ethical conduct of the study

The study will be carried out in accordance with the World Medical Association Declaration of Helsinki (1964) and its revisions (Tokyo [1975], Venice [1983], Hong Kong [1989], South Africa [1996] and Edinburgh [2000]).

Favourable ethical opinion will be sought from an appropriate REC before patients are recruited to this clinical trial. Patients will only be allowed to enter the study once they have provided written informed consent.

The Chief Investigator, Dr Malcolm Sim will be responsible for updating the Ethics committee of any new information related to the study.

14. INSURANCE AND INDEMNITY

This study is sponsored by NHS Greater Glasgow and Clyde. The sponsor will be liable for negligent harm caused by the design of the trial. The NHS has a duty of care to patients treated, whether or not the patient is taking part in a clinical trial, and the NHS remains liable for clinical negligence and other negligent harm to patients under its duty of care. NHS indemnity is provided under the Clinical Negligence and Other Risks Indemnity Scheme (CNORIS).

15. FUNDING

The work has been funded by an unrestricted research grant and equipment from Aerogen Limited.

16. ANNUAL REPORTS

Annual reports will be submitted to the ethics committee and sponsor with the first submitted one year after the date that all trial related approvals are in place.

17. DISSEMINATION OF FINDINGS

The data from this study will be presented at critical care meetings and published in peer reviewed critical care journals as well as help to inform best practice for nebulisation on saline in ventilated patients with secretions in Intensive Care.

18. REFERENCES

1. Terzi N., Guerin C. And Goncalves M. What's new in management and clearing of airway secretions in ICU patients? It is time to focus on cough augmentation. *Intensive Care Medicine* 2019;**45:**865-868.
2. Jaber S., Quintard H. And Cinotti R.et al. Risk factors and outcomes for airway failure versus non-airway failure in the intensive care unit: a muticentre observational study of 1514 extubation procedures. *Critical Care* 2018;**22:**236.
3. Thille A.W., Richard J.C. and Broachard L. The decision to extubate in the intensive care unit. *Am. J. Respir. Crit. Care Med.* 2013;**187(12):**1294-302.
4. Keal, E. and Reid, L. The neuraminic acid content of sputum in chronic bronchitis. *Thorax* 1972; **27**:643.
5. Lopez-Vidriero M.T., Charman J., Keal E. and De Silva D.J. Sputum viscosity: correlation with chemical and clinical features in chronic bronchitis. *Thorax* 1973; **28**:401.
6. Julious SA. Sample size of 12 per group rule of thumb for a pilot study. *Pharmaceutical Statistics* 2005; **4(4):** 287–291.
